# A cross-sectional study design to assess the sexual experiences and contraceptive use of adolescents and youths attending high school and college in Jimma town

**DOI:** 10.1186/s40834-022-00174-z

**Published:** 2022-06-01

**Authors:** Gudina Terefe Tucho, Netsanet Workneh, Mubarek Abera, Jemal Abafita

**Affiliations:** 1 Department of Environmental Health Sciences and Technology, Institute of Health, Jimma University, Jimma, Ethiopia; 2 Department of Pediatrics, Institute of Health, Jimma University, Jimma, Ethiopia; 3 Department of Psychiatry, Institute of Health, Jimma University, Jimma, Ethiopia; 4 Department of Economics, College of Business and Economics, Jimma University, Jimma, Ethiopia

**Keywords:** Adolescents and youths, Sexual and reproductive health, Contraceptives, HIV/AIDS, Pregnancy

## Abstract

**Background:**

Adolescents and youths in Ethiopia have limited access to reproductive health services designed to address their needs. Therefore, the study aims to assess adolescents' and youth’s sexual practice, contraceptive use, and behavioral patterns towards safe sexual exercise.

**Methods:**

A quantitative cross-sectional study design was used on 374 students selected from high school and vocational colleges to assess their sexual experience and contraceptive use and related perceptions using pre-tested self-administered semi-structured questionnaires. We used descriptive analyses to report their sexual and reproductive health status and logistic regression to examine the association between contraceptive use and other variables.

**Results:**

The results show that 52.7% of the respondents (students) migrated or moved from rural to urban to continue their high school and college education at urban. 41.7% of the respondents were with less than 18 years, of which 75.9% of them already initiated sexual intercourse. Of all the students who started sexual intercourse (51.1%), only 30.9% used contraceptives, but condom use accounts for 49.2%. Overall, 84.8% of those who practiced sexual intercourse were at risk of acquiring sexually transmitted diseases due to not using a condom. About 3% of the respondents reported unwanted pregnancy experiences, of which 64% of these pregnancies were reported to be aborted.

**Conclusion:**

Adolescents and youths attending high schools and College are at increased risk of acquiring HIV and unwanted pregnancy due to low levels of contraceptive use. Specifically, a designed youths-friendly reproductive health service is needed to avert related problems and contribute to sustainable development goals.

## Background

Approximately half of the world’s population is under the age of 25 years, of which 1.8 billion are young people aged between 10 and 24 years. A majority (90%) of these young people live in low- and middle-income countries (LMICs), where poverty and unemployment are most prevalent. In Africa, this population represents over a third of its people, making it the youngest globally. In particular, sub-Saharan Africa is the only region of the world where its young people continue to grow substantially. Adolescents are part of this group of population spanning the period from 10 to 19 years of age. The situation in Ethiopia is not different. Ethiopia is a very young nation with about 63% of its population under 25 years. Young people aged 10–24 years constitute about 33% of the total population, with nearly 22% being adolescents aged between 10 and 19 years [1]. This age represents a transitional stage of physical and psychological human development.

Young people are vital to the economic, political, and social development of Africa. They are indispensable in terms of accelerating economic growth and reducing poverty. Adolescents, in particular, are the future family and working forces expected to contribute to societal developments. Investing in adolescents' and youths' wellbeing would have far-reaching implications for ensuring Africa’s development. Consequently, there is a strong rationale to develop appropriate policies and strategies that accommodate the needs of this crucial segment of the population. However, adolescents and young people face unique vulnerabilities and challenges that are often unaddressed by policies and interventions. The sexual and reproductive health needs of adolescents remain unmet, mainly due to lack of knowledge, social stigma, laws, and policies preventing the provision of contraception and/or abortion to unmarried adolescents (or any adolescent in some cases), and judgmental attitudes among service providers [2]. Decisions made during adolescence, particularly regarding sexual and reproductive health, can have a long-term impact on the young person and human development in general. The rapidly evolving physical, intellectual, and emotional development during this crucial life course makes adolescent health care challenging. Many adolescents die prematurely due to preventable illnesses associated with reproductive health. The majority of non-communicable diseases and misbehaviors like substance misuse have their roots in adolescence.

The disadvantage and vulnerability of women to many of the health problems, including abnormality to their children, frequently have also rooted in adolescent behavioral patterns [3], risks for HIV infection and other non-communicable diseases like cancer [4]; and risks for maternal and child health [5, 6]. The adolescent population faces multifaceted and interrelated social, economic, and health problems, among which risks to their reproductive health are the major ones. In particular, girls face many challenges during early adolescence, such as unwanted sex, high chances of unwanted pregnancies, unsafe abortions, and sexually-transmitted infections, including HIV. The implications of these challenges are often severe, with consequences jeopardizing other areas of health, education, welfare, and future opportunities during later stages of their lives.

Adolescents in Ethiopia have limited access to reproductive health services designed to address their particular needs specifically. Inadequate knowledge about adolescent sexual behavior, cultural influences, limited access to reproductive health information, and lack of adolescent and youths-friendly sexual and reproductive health is common among adolescents [1, 7, 8]. Neither the health service nor the country's education system adequately considers the sexual and reproductive issues of this young segment of the population. Generally, there are limited recent data regarding adolescent health in Ethiopia. Regarding reproductive health problems, teenage pregnancy is associated with several health and social issues; both mothers and children are at increased risk of morbidity and mortality. According to Ethiopian DHS 2016, 13 percent of women start childbearing at 15—19 [9]. In particular, female youths face many risks, including HIV/AIDS infection, sexual harassment, and teenage pregnancy. Female adolescents are also more likely to have limited access to essential social services such as education and HIV/SRH related services [1, 7, 10].

Having limited access to sexual and reproduction services imposes adolescents and youth to HIV infection. For instance, globally, by the end of 2018, about 38 million people were living with HIV, which Sub-Saharan Africa accounts for an estimated 71% of the global burden. In Ethiopia, an estimated 715 thousand people lived with HIV in 2015, which increased to 722 thousand in 2017 [11]. The current prevalence of HIV infection in Ethiopia shows inconsistent trends across the country, with the highest prevalence of up to 4.8% in some regions [11].

A global report shows between 2015–19, and there were 121 million unintended pregnancies annually, corresponding to a worldwide rate of 64 unintended pregnancies per 1000 women aged 15–49 years, of which 61% ended in abortion, corresponding to a global abortion rate of 39 per 1000 women [12]. The overall unintended pregnancy prevalence rate in Sub-Saharan Africa ranges from 10.8% in Nigeria to 54.5% in Namibia, including women in 15–19 years [13, 14], and related high level of unsafe abortion [15]. A study report from Addis Ababa among reproductive age groups shows that 37.8% had unwanted pregnancies, of which 39.6% had induced abortion at some point in their lives[16]. The prevalence of teenage pregnancy in different regions of Ethiopia varies from 7.7—30.2% among the age group of 16– 19 years old, which is associated with low contraceptive use, not knowing fertile period in the menstrual cycle, and a low level of awareness among others [17–20]. These studies highlighted the lack of programs that encourage teenage reproductive health issues.

Ethiopia has made many efforts to control the HIV/AIDS transmission and psychosocial effects and end the AIDS epidemic by 2030 [21]. Antiretroviral Therapy (ART) is mainly considered to slow the disease's progress and improve the patients' health [22, 23]. Since the first global treatment target was set in 2003, annual AIDS-related deaths have decreased by 43% globally and 36% in Sub-Saharan Africa [21, 24]. However, still, a lot of challenges are ahead due to increasing new HIV infections. In 2015 alone, about 1 million new HIV infections were occurred in eastern and southern African countries [21].

Moreover, adolescents and youths face many challenges, such as early pregnancy, difficulties accessing contraception, and high HIV and sexually transmitted infections [25]. However, many of the current intervention strategies fail to provide young people with supportive and youth-friendly services. Interventions specifically designed and targeting adolescents and youths are crucial to curb the problems [26], however, overlooked in many countries. Many of the studies conducted in Ethiopia mainly focuses on married women in the reproductive age groups and teenage pregnancy and abortion. Studies focusing on adolescents and youth sexual experience and contraceptive use are not well understood. However, they are essential to tackle related unwanted pregnancy, unsafe abortion, and infection of HIV.

Moreover, devising an appropriate intervention strategy requires a better understanding of the youth’s and adolescent’s current sexual and reproductive status and awareness level. Therefore, the study aims to assess adolescents' and youth’s sexual practice, contraceptive use, and behavioral patterns towards safe sexual exercise. The study results are vital to indicate the risks of HIV infection, unwanted pregnancy, unsafe abortion, and the possibility of achieving related sustainable development goals.

## Methods

### Study area, design, and period

This study was conducted in Jimma, the main town of Jimma zone, one of Oromia Regional State zones known for its coffee. Jimma city is a connection to most of the towns in the South-western parts of the country. The city is home to multiple ethnic groups, of which the Oromo represent the largest group. Jimma University, a public higher education institute (HEI), is located at the heart of the city and accommodates over 35,000 students (mainly adolescents and youths) from all over the country. Besides the university's student population, the city is home to many adolescents from the town and its surroundings attending schools in private and government training institutions (colleges and vocational schools). The city has two public hospitals, two private hospitals, four public health centers, and several private healthcare facilities and specialty organizations working on reproductive healthcare services. Approximately over fifty thousand adolescents and youths live in the town. Many of the students are living separated from their families under limited parental follow-up. The city is one of the areas where “Khat” production and consumption are widely practiced in the general population. A school-based cross-sectional study was conducted in April 2018 on adolescents and youths between 16 and 25 years attending public high schools and colleges (private and government) in the town.

### Study population and sample size determination

The study focuses on the vulnerable section of the population, specifically adolescents and youths attending high schools and vocational colleges. The town has about ten high schools, three vocational colleges, and two universities. This study purposely selected one high school nearer to the university and two colleges, one from each public and private College, by considering the diversity of the students and likelihood of risks. Accordingly, the study population was selected from Jiren high school, Rift valley University Jimma branch, and Jimma teachers training College. The sample size was determined based on a single population proportion formula with a 95% confidence interval, margin of error of 5% [27], and a population proportion of 33%, a prevalence of emergency contraceptive use among university students in Ethiopia [28]. Three hundred seventy-four students were considered for the study by adding a 10% non-response rate for compensation from high schools and colleges. The study purposively regarded an equal number of students (125) from high school, private and public colleges. These were selected randomly selected classes until the required number of participants are considered. According to their language preferences, the study participants from different classes were considered for self-administered questionnaires (Afan Oromo or Amharic). The study participants were given the full protection of confidentiality and privacy to respond by avoiding personal identifiers from the questionnaires.

### Data collection and analysis

The sexual and reproductive health status were investigated based on self-reported responses on sexual and reproductive health-related questions adapted from WHO standardized tools [29]. Accordingly, the self-reported level of reproductive health indicators related to the initiation of sexual intercourse, use of contraceptives, knowledge of contraceptives, teenage pregnancy, abortion, sexual harassment, and other related issues were examined based on the obtained information. In Ethiopia, only opposite sexual relations are officially and culturally recognized; thus, our analysis only considered opposite sexual relations. The data was collected by using pre-tested self-administered semi-structured questionnaires. The questionnaires were prepared in English, translated to local languages (Afan Oromo and Amharic), and translated back to English to better understand. The pre-test was done on 5% of the population in other schools and colleges as a pilot study to avoid ambiguity. The quality of the data was assured by assigning well-qualified data collectors and site supervisors. Trained supervisors were assigned to correct any encountered problems and check for completeness and consistency at the end of each data collection day. Additional data checking and editing were performed before analysis.

Prior to data collection, an ethical approval letter was obtained from the institutional review board (IRB) of Jimma University Institute of health (Ref: IHRPGD/3005) on 26/01/2018. The study ensured the participants' confidentiality and anonymity throughout the data collection, analysis, and report. The participants provided their written informed consent to participate in this study. Moreover, the participants were informed about declining/participating at any stage of the data collection process if they are not interested in the study. Permission to conduct the study was sought in writing from the selected schools' local authorities and administrative units.

The data were analyzed by using a statistical software package (SPSS V.21). Prior to analysis, the data was checked for its completeness and consistency. First, a descriptive frequency distribution of the data was presented to check its normal distribution with subsequent outlier removals. The chi-square test was performed to identify the association between categorical variables of interests. Variables showing significant association in the chi-square tests were considered for logistic regression to determine the association of their strength. The use of contraceptives refers to modern contraceptive methods during any sexual intercourse, but we emphasized first-time and casual sexual intercourse. Accordingly, participants were asked whether they use modern contraceptives with yes or no choices where yes has given the value of 1 and no the value of 0.

## Results

### Sociodemographic conditions of the study participants

The sociodemographic characteristics of the study participants are presented in Table 1. As shown in the table, 70.9% of the study participants are from the Oromo ethnic group, followed by Amhara (10.7%). The study participants were from different religious groups, of which 36.6% are from Islam, 32.9% from Orthodox Christianity, and Protestants with 27.8%. The participants were from high school and vocational college, of which 41.7% of them are under 18 years, followed by 33.4% between the age of 19 – 21 years, and only 3.7% were above 24 years of age. The proportion of male and female study participants was 51.1% and 48.9%, respectively. 33.7% of the study participants were from high school, and 66.3% were from private and government colleges. Married study participants were accounted for 11.8%.

**Table 1:** Sociodemographic conditions of the study participants (*n* = 374)

Items	Category	Frequency (yes)	Percent (%)
Ethnicity	Oromo	265	70.9
	Amhara	40	10.7
	Dawuro	35	9.4
	Tigire	11	2.9
	Others^a^	23	6.1
Religion	Islam	137	36.6
	Orthodox	123	32.9
	Protestant	104	27.8
	Traditional/waqeffataa	10	2.7
Age (years)	< = 18	156	41.7
	19–21	125	33.4
	22–24	79	21.1
	> 24	14	3.7
Sex	Male	191	51.1
	Female	183	48.9
Educational level	Second cycle (9–10)	126	33.7
	College (10 + 2)	248	66.3
Marital status	Married	44	11.8
	Single	327	87.4
	Divorced	3	0.8

Others^a^ = Gurage, Yem, kefa, etc.

The socio-economic conditions of the student’s (participant’s) families were presented in Table 2. Accordingly, more than half (52.7%) of the students were from rural areas. Half of the students were from families having a family size of 6 – 10 persons. Moreover, 83% of the rural students lived in rented rooms based-on their family’s financial support.

**Table 2:** Socioeconomic conditions of the students and their families (*n* = 374)

Items	Category	Frequency (yes)	Percentage (%)
Family residence	Rural	197	52.7
	Urban	177	47.3
Family size	< = 5 members	179	47.9
	6–10 members	188	50.3
	> 10 members	7	1.9
Students’ source of income	Family	311	83.2
	Salary	20	5.3
	Own business	27	7.2
	Others sources^a^	16	4.3

Other sources^a^ = living with relatives, etc.

### Sexual and reproductive health conditions of the study participants

Table 3 shows the participant’s sexual experiences and conditions of contraceptive use. Accordingly, 51.1% of the respondents reported as they initiated sexual intercourse. 61.8% of the respondents who started sexual intercourse responded as they were forced by a partner or forced their partner to have sexual intercourse. Moreover, 75.9% of the participants initiated sexual intercourse at the age of less than 18 years, of which the age of less than 15 years represents 28.9%. The results show that only 30.9% of those who initiated sexual intercourse used any form of contraceptives to avoid pregnancy. Among these, modern contraceptive use (condom and oral pills) represents 62.8%. The remaining was natural methods (safe menstrual period and coitus withdrawal), where the use of Condoms accounts for 49.2% of the modern contraceptives used.

**Table 3:** The students’ sexual relationship and contraceptive use

Item	Description	Frequency	Percent
Did you have an opposite-sex relationship?	Yes	191	51.1
	No	183	48.9
	Total	374	100.0
What was your reason for having sexual relation?	Forced partner to have sex	26	13.6
	Forced by the partner to have sex	92	48.2
	We both were equally willing	73	38.2
	Total	191	100
What was your age at first-time sex?	Less than 15 years	42	22.0
	16 – 18 years	103	53.9
	19 – 21 years	41	21.5
	22 – 24 years	5	2.6
	Total	191	100.0
Did you use any contraceptives for first-time sex?	Yes	59	30.9
	No	132	69.1
	Total	191	100.0
Which contraceptive method did you use?	Condom	29	49.2
	Oral pills	8	13.6
	Safe menstrual period and withdrawal	22	37.3
	Total	59	100.0

### Perception of the students towards contraceptive use

Participants were asked to respond with their view of contraceptives' benefits and related consequences for not using (Table 4). Accordingly, when they were asked about the possibility of getting pregnant on the first-time sex, only 130 (34.8%) were sure about it, 91 (24.3%) responded no risk of getting pregnant, and the remaining 153 (40.9%) of them were hesitant. In addition, only 158 (42.2%) knows the chances of getting pregnant during the ovulation period (halfway of menstrual period), 120 (32.1%) were not sure, and 96 (25.7%) responded as a woman cannot get pregnant during her ovulation period. Moreover, 35% of the respondents either do not know or were not sure about using a condom to prevent unwanted pregnancy and sexually transmitted diseases. Furthermore, 11 (2.9%) of the respondents reported the experience of pregnancy, of which 7 (63.7%) of them reported termination (abortion).

**Table 4:** Perception of the study participants towards contraceptives use

Questions	Response	Frequency	Percent
Can a woman become pregnant on first-time sexual intercourse?	yes	130	34.8
	no	91	24.3
	not sure	153	40.9
	Total	374	100.0
Can a woman get pregnant if she has sex halfway between her menstrual period?	yes	158	42.2
	no	96	25.7
	not sure	120	32.1
	Total	374	100.0
Do you think that condoms prevent pregnancy and other diseases?	yes	243	65.0
	no	74	19.8
	not sure	57	15.2
	Total	374	100.0
Have you ever been pregnant?	yes	11	2.9
	no	363	97.1
	Total	374	100.0
	Currently pregnant	1	9.1
	Aborted at clinics	3	27.3
What happened to the pregnancy?	self-abortion	4	36.4
	Gave live-birth	3	27.3
	Total	11	100.0

### Factors determining sexual practice and use of contraceptives

Several factors can determine the adolescent’s and youth’s behavior towards their sexual and reproductive health activities. Some of these factors are shown in Table 5 based on the logistic regression results. In this study, 191 (51%) of the participants had reported their sexual intercourse experience, of which 51 (30.9%) used contraceptives during intercourse. Out of those using contraceptives, about 50% of them used condom, 8 (13%) use oral pills and the remaining used natural methods. Regarding the level of education, being in College (10 + 2 years) was associated with an increasing level of use of contraceptives [AOR: 0.625, 95% C.I. 0.527 – 0.760], compared with Second Cycle (9–10 years). This implies that an increase in the level of education decreases the practice of unsafe sexual intercourse. The study participants' age was also another factor that showed strong statistical significance associated with the initiation of casual sexual practice [AOR: 0.341, 95% C.I. 0.256 – 0.455]; an increase in the students' age decreases the practice of casual sexual practice. Knowledge of the possibility of getting pregnant on first-time sex and during the ovulation period showed a statistically significant association with contraceptives (see Table 5). Family residence increases the likelihood of initiation of sexual intercourse by about 1.5 times [AOR: 1.499, 95% C.I. 0.996 – 2.254] although statistically not significant. Moreover, knowing the benefits of condoms increases contraceptives' likelihood more than two times [AOR: 2.01, 95% C.I. 1.235 – 3.269].

**Table 5:** Factors determining adolescents and youths on having opposite casual sexual intercourse and use of contraceptives using Adjusted Odds Ratio (AOR)

		Sig	AOR	95% C.I. for AOR	
				Lower	Upper
Having opposite casual sexual relation	Educational level	< 0.001	0.627	0.517	0.760
	Student’s age	< 0.001	0.341	0.256	0.455
	School sexual and reproductive health service	< 0.050	1.511	0.961	2.376
	Parent’s schooling support	< 0.500	1.259	0.665	2.382
	Family residence	0.050	1.499	0.996	2.254
Use of contraceptives	Age of the students	< 0.005	0.587	0.402	0.856
	Knowledge of getting pregnancy half-way between menstrual cycle	< 0.001	1.675	1.312	2.139
	Knowledge of getting pregnancy on fist-time sex	< 0.025	1.552	1.063	2.265
	Educational level	< 0.050	0.369	0.145	0.940
	Knowledge of the benefits of condom	< 0.005	2.010	1.235	3.269

## Discussion

This study evaluated adolescents and youths' sexual and reproductive health conditions attending high school and vocational colleges. It was shown that 41.7% of the study participants were less than 18 years. Over 50% of the study participants have practiced sexual intercourse, of which 75.9% of them initiated their first-time sex at the age of less than 18 years (Table 3). Moreover, 61.8% of them started sexual intercourse were either enforced by their partner or enforced their partner to have sexual intercourse against their will. Both conditions (earlier age and partner pressure) could compromise these groups' decision-making to avoid casual sexual intercourse and risky sexual behaviors.

Moreover, only 30.9% of the youths who practiced sexual intercourse used any form of contraceptive, of which condom use accounts for 49.2%. The prevalence of condom use is comparable (49%) with the study report from two rural high schools in North-Western Ethiopia [30] and lower than the study report from Jijiga university (59.6%) [31] and Haramaya University (55.7%) [32]. Overall, 84.8% of those who practiced sexual intercourse were at risk of acquiring any form of sexually transmitted diseases due to not using a condom. In addition, 65.2% and 57.9% of the study participants do not know the possibility of getting pregnant on the first-time of sex and during the ovulation period. In addition, 35% of the study participants do not know the benefits of condoms to prevent unwanted pregnancy and sexually transmitted diseases. These risky sexual practices accompanied by a low level of awareness could lead to unwanted pregnancy and sexually transmitted diseases like HIV/AIDS.

About 3% of the study participants reported a history of pregnancy, 63.7% reported termination. This is comparable with the study report from Aletawondo high School of Sidama Zone in Ethiopia, where 82% of the students with unwanted pregnancies had reported termination [33]. This is a big concern that unwanted pregnancy and subsequent abortion can contribute to adolescent and youth pregnancy-related health complications and deaths. Literature reports show that early initiation of sexual practice, early marriage, and pregnancy has shown significant association with commercial sex work practice and HIV/AIDS infections [34, 35]. Giving live-birth at adolescence ages will also have many socioeconomic and psychosocial consequences resulting in early life crises these groups cannot shoulder. It is shown that unwanted pregnancy and unprotected sex leads to several socioeconomic problems such as homelessness, unemployment, drop-in education, and commercial sex work practices [36, 37].

Moreover, 52.7% of the study participants migrated from rural areas to attend their urban schools, relying on their families for their basic needs and schooling. The majority of these students live in rented houses without family follow-up. Moreover, many of these youths are from large low-income families with insufficient capacity to provide them enough money for their basic needs. Inability to fulfill their basic needs by their family’s support may force them to look for an additional source of income. An extra income source such as paid job could be hard to find, but students may not have spare time to work while studying. These conditions may enforce them to go for risky jobs such as transactional sex, which involves an exchange of gifts for sex. Rural to urban migration by itself has a lot of socioeconomic and health consequences such as high-risk sexual behavior, HIV infection, and transactional sex [25, 38, 39]. Young girls mostly deprived of economic access, difficulty accessing school, and having social pressure are at risk of transactional sex [26]. Early initiation of sexual practice was also reported to have a significant association with commercial sex work [40, 41]. Women involved in risky sexual relations have many psychosocial and health problems that could lead them to illegal migration to other countries.

Furthermore, the inability to take immediate and sustainable adolescent and youth-friendly action will have many policy implications. For instance, youths' and adolescents' reproductive health is directly related to Sustainable Development Goals 3,4, and 5. Some of the targets of these goals focus on reducing maternal and child mortality rates, preventing HIV/AIDS, providing equitable quality education for all boys and girls, and eliminating trafficking and sexual exploitation against girls. However, the current intervention strategy mainly focuses on curative aspects such as ART provision to HIV patients rather than providing comprehensive reproductive health services [23, 42]. As a strategy to end HIV/AIDS by 2030, Ethiopia adopted the global 90–90–90 HIV prevention targeting testing, treatment, and reduction of viral loads by 90% [23]. However, new HIV infections started to increase in the last few years despite growing ART services and a decline in HIV/AIDS-related deaths. These could be attributed to reducing sexual and reproductive health awareness creation and increasing risky sexual practices. Risky sexual practices inevitably involve unwanted teenage pregnancy entailing school dropout, abortion, homelessness driven by sociocultural pressure and pregnancy related health complications. Hence, without addressing these issues achieving the aforementioned sustainable development goals cannot be possible.

Adolescent’s sexual and reproductive health is a global challenge but overlooked in many countries, including Ethiopia. Some of the challenges adolescents face include early pregnancy, difficulties accessing contraception, and high HIV and sexually transmitted infections [43]. However, many intervention strategies are neglected nowadays to provide adolescents and youths with supportive and youth-friendly awareness creation and basic reproductive health services. Interventions specifically designed and targeting adolescents and youths are very crucial to curb reproductive health-related problems.

## Conclusion

Adolescents and youths are a vital population category requiring special attention. They need continuous awareness creation and follow-up regarding their sexual and reproductive health. Otherwise, they are highly vulnerable to unsafe sexual intercourse, unwanted pregnancy, unsafe abortion, and HIV infection. Exposing them to these issues will have many health, psychosocial, socioeconomic, and career consequences. However, currently, the government is not focusing on creating awareness and devising a youth-friendly policy benefiting them. This led to a low level of understanding and protection during sexual intercourse. This is evidenced in the current study, where most students reported unsafe sex practices due to a low level of contraceptive use. The majority of the adolescents started sexual intercourse at an early age without any protection. Furthermore, many study participants do not know the benefits of contraceptives and condom use against unwanted pregnancy and sexually transmitted diseases. Therefore, in the absence of adolescents and youth’s friendly preventive strategy, achieving sustainable development targets related to HIV/AIDS, reducing maternal mortality rate, decreasing abortion-related health complications, increasing women empowerment, and providing equal educational access to girls is not possible.

## Data Availability

Data protection, confidentiality, and privacy of the data have been anonymized from the beginning of data collection and available from the corresponding (no personal identifications were used).

## Abbreviations

HIV: Human Immuno Virus
AIDS: Human Immuno Deficiency Syndrome
